# SLEEP APNEA ASSOCIATED COMORBIDITIES AND MORTALITY IN AN OLDER VETERAN POPULATION

**DOI:** 10.1093/geroni/igz038.982

**Published:** 2019-11-08

**Authors:** Denise M Kresevic, muralidahar pallaki, Christopher J Burant, Clare Gideon, Emily Schroeder, Peijun Chen

**Affiliations:** 1 Northeast Ohio VAMC, Cleveland, Ohio, United States; 2 Northeast Ohio va health system, Cleveland, Ohio, United States; 3 Case Western Reserve University, Cleveland, Ohio, United States

## Abstract

Evidence continues to mount that sleep apnea (SA) occurs in 10-25% of Americans and is associated with significant morbidity and mortality (Schulman 2018). Among veterans, SA has been reported four times more often as compared to other non-veteran cohorts. (Wong 2015). The risk of developing dementia is increased in older individuals with OSA (Shastri, Bangar, & Holmes, 2015). The prevalence and characteristics of older adults with dementia and sleep apnea is not well known and long-term population-based studies on mortality have been lacking. Recent studies have reported overall mortality rates of 19%, in those individuals with SA, an increased rate of 1.5-3 times the mortality rate as compared to those individuals those without SA. Current recommendations support SA screening of high risk individuals including those with symptoms of snoring, fatigue, memory and concentration problems and mood changes. (Krist 2018). Despite a large number of older adults with suspected SA and comorbidities, the majority are not screened, referred, diagnosed and treated. In this VA pilot study of outpatient older male veterans with dementia and SA, N=195, mean age 75.83 years, SD=9.1, 51.3% were white, 37.5% were black. Frequently found comorbidities were: hypertension 88%, congestive heart failure 41%, Diabetes. 62% and, stroke 21%. Of note, among those who died, SA was significantly related to congested heart failure (r=.32, p<.001) and COPD (r=.40, p<.001). The overall mortality rate of 27% was higher than previous reports. Further investigation is needed to better understand the relationship between comorbidities, and SA, screening, treatment and mortality.

